# Chronic social stress in pigs impairs intestinal barrier and nutrient transporter function, and alters neuro-immune mediator and receptor expression

**DOI:** 10.1371/journal.pone.0171617

**Published:** 2017-02-07

**Authors:** Yihang Li, Zehe Song, Katelyn A. Kerr, Adam J. Moeser

**Affiliations:** 1 Gastrointestinal Stress Biology Laboratory, Michigan State University, East Lansing, Michigan, United States of America; 2 Department of Large Animal Clinical Sciences, College of Veterinary Medicine, Michigan State University, East Lansing, Michigan, United States of America; 3 Zhejiang University, College of Animal Sciences, Hangzhou, P. R. China; 4 Neuroscience Program, Michigan State University, East Lansing, Michigan, United States of America; 5 Department of Physiology, Michigan State University, East Lansing, Michigan, United States of America; University of California Los Angeles, UNITED STATES

## Abstract

Psychosocial stress is a major factor driving gastrointestinal (GI) pathophysiology and disease susceptibility in humans and animals. The mechanisms governing susceptibility to stress-induced GI disease remain poorly understood. In the present study, we investigated the influence of chronic social stress (CSS) in pigs, induced by 7 d of chronic mixing/crowding stress, on intestinal barrier and nutrient transport function, corticotropin releasing factor (CRF) signaling and immunological responses. Results from this study showed that CSS resulted in a significant impairment of ileal and colonic barrier function indicated by reduced transepithelial electrical resistance (TER) in the ileum and increased FD4 flux in the ileum (by 0.8 fold) and colon (by 0.7 fold). Ileal sodium glucose linked transporter 1 (SGLT-1) function, measured as glucose-induced changes in short-circuit current (*I*_sc_), was diminished (by 52%) in CSS pigs, associated with reduced body weight gain and feed efficiency. Although reductions in SGLT-1 function were observed in CSS pigs, mRNA expression for SGLT-1, villus heights were increased in CSS pigs. Corticotropin releasing factor (CRF) mRNA was upregulated (by 0.9 fold) in the ileum of CSS pigs but not in the colon. Urocortin 2 (Ucn2) mRNA was upregulated (by 1.5 fold) in the colon of CSS pigs, but not in the ileum. In CSS pigs, a downregulation of pro-inflammatory cytokines mRNA (IL1B, TNFA, IL8, and IL6) was observed in both ileum and colon, compared with controls. In contrast CSS induced a marked upregulation of mRNA for IL10 and mast cell chymase gene (*CMA1*) in the ileum and colon. Together, these data demonstrate that chronic stress in pigs results in significant alterations in intestinal barrier and nutrient transport function and neuro-immune mediator and receptor expression.

## Introduction

Environmental and psychosocial stressors play a central role in the initiation and (or) exacerbation of common and burdensome intestinal disorders of humans and animals. In humans, dysregulated stress responses are an important component in the pathophysiology of diseases such as irritable bowel syndrome (IBS) [1–3] and the inflammatory bowel diseases (IBD) [4, 5]. In agriculturally-important species such as the pig, psychosocial and environmental stressors associated with production practices (e.g., weaning, mixing/crowding stress and heat stress have significant deleterious impacts on performance and GI function [6–8] and increased susceptibility to infectious GI diseases [9]. While the negative impacts of stress in GI disorders is well established, targeted therapies for stress-induced GI disorders are lacking thus highlighting the need for a more fundamental understanding of stress-induced GI disease mechanisms in people and animals.

There has been a large number of rodent research models developed to study the underlying mechanisms of stress-induced GI disease. Diverse stress paradigms in rodents (e.g. restraint stress, water avoidance stress, neonatal maternal separation, etc.) have been shown to impair barrier functions of the GI epithelium, enteric nervous system (ENS), and immune system. There are significant effects on stress on the GI epithelial barrier reported across several models and in humans including increased intestinal permeability, ion transport and hypersecretion, and mucus secretion [10–12]. Several investigations in rodents and pigs have demonstrated the influence of stress on ENS phenotype and function is greatly influenced by stressors indicated by significant alterations in GI motility patterns, increased secretomotor function, and heightened sensitization of visceral afferent neurons and visceral motor responses [13–16]. Studies in multiple species have demonstrated diverse effects of stress on the GI immune system. In rodent studies, chronic stressors such as crowding/mixing stress and chronic subordinate housing were shown to enhance GI inflammation [17–19]. Early life stress models such as neonatal maternal separation (NMS) stress in rodents were shown to induce a pro-inflammatory state and increases susceptibility to colitis [10, 20]; however, in contrast, early weaning stress in pigs resulted in suppressed innate immune responses to a later life challenge with enterotoxigenic *E*. *coli* [9]. Activation of intestinal mast cells have been shown in multiple animal models and in humans to be a key immune player in stress-induced GI disease [11, 21, 22].

Several important neuro-immune mechanisms have been identified to play a major role in mediating stress-induced alterations in GI function. The most-well studied system regulating the central and GI stress responses is the corticotropin releasing factor (CRF) system. The CRF system is composed of CRF and the related family of urocortins 1–3 (Ucn) and their G-protein coupled CRF receptors CRF_1_ and CRF_2_, all of which are expressed in the central and peripheral nervous systems and on multiple cell types within the GI system including enteric neurons, immune cells and the epithelium [23, 24]. The CRF system has been shown to mediate a wide range of stress-induced GI changes including intestinal permeability [16, 25], hypersecretion [15, 26], motility disturbances [27, 28], inflammation [29, 30] and visceral hypersensitivity [31, 32]. The CRF receptor subtype plays an important role in dictating the GI stress response with activation of CRF_1_ pathways mediating increased colonic motility and visceral hypersensitivity while CRF_2_ plays an opposing, protective role [27, 32, 33]. Many of the CRF and stress-induced disturbances in GI function including increased intestinal permeability, mucin and ion secretion, and visceral hypersensitivity are mediated via intestinal mast cell degranulation [8, 11], highlighting the importance of the CRF-mast cell axis in the GI response to stress.

Together, the scientific literature demonstrates the profound and diverse impacts of stress on GI function and disease susceptibility. The predominance of research in the field of stress-induced GI pathophysiology has been conducted in rodent models. In the present study, we investigated the influence of chronic social stress on GI barrier function and neuro-immune responses in pigs as a biomedical and agriculturally-relevant model for stress-related GI diseases in humans and animals, respectively.

## Materials and methods

### Animals and experimental design

This study was carried out in strict accordance with the recommendations in the Guide for the Care and Use of Agricultural Animals in Research and Teaching. All animal protocols were approved by the North Carolina State University Animal Care and Use Committee (09-047-B) prior to the experiment. A total of 48 Yorkshire-cross barrows (9 wks of age and 22.1 ± 0.54 kg), obtained from the NC State University Swine and Teaching Unit, were used in this experiment. Upon arrival, pigs were weighed and blocked by body weight, then randomly assigned to one of 2 experimental groups: control or mixing/crowding stress. During the first week (d 1–7) after arrival, pigs were housed 4 pigs/pen at the recommended [34] stocking density (0.38 m^2^ floor space/pig) in a standard temperature-controlled, commercial-style facility to acclimate to the pens. Food and water was provided *ad libitum* and formulated to meet the nutrient requirements for this age of pig [35]. After the 7 d acclimation period, chronic social stress (CSS) was induced moving individual pigs from their acclimation pen to a new pen with three unfamiliar pigs who were also from different acclimation pens (n = 6 pens, 4 pigs per pen). As an additional stress, pigs within the CSS group were housed with reduced floor space allowance (0.17 m^2^ floor space/pig) after mixing. The control groups remained undisturbed in their acclimation pens undisturbed. Animal body weight and feed intake per pen (n = 6 per treatment) were recorded on d 1, 8 and 14. No difference of initial body weight was observed. Average daily gain (ADG), average daily feed intake (ADFI) and feed:gain ratio (F:G) of day 8 to 14 were calculated.

### Ussing chamber experiment

At the end of the trial, n = 6 pigs per experimental group were euthanized for sample collection. To minimize the stress during euthanasia, pigs were sedated using a combination of Telazol^®^ (3 mg/mL), Ketamine (7.5 mg/kg), and Xylazine (7.5 mg/kg). Following sedation, pigs were euthanized with Euthanasia solution (pentobarbital sodium, 86 mg/kg). Immediately following euthanasia, segments of distal ileum and ascending colon were prepared for Ussing chamber experiments as described previously [36]. Briefly, the mucosa was stripped from the seromuscular layer in oxygenated (95% O_2_, 5% CO_2_) Ringer’s solution. Tissues were then mounted in 1.13-cm^2^-aperture Ussing chambers. Tissues were bathed on the serosal and mucosal sides with 10 mL of oxygenated Ringer’s solution maintained at 37°C. The spontaneous potential difference (PD) was measured using Ringer-agar bridges connected to calomel electrodes, and the PD was short-circuited through Ag-AgCl electrodes using a voltage clamp that corrected for fluid resistance. Tissues were maintained in the short-circuited state, except for brief intervals to record the open-circuit PD. Transepithelial electrical resistance (TER; Ω•cm^2^) was calculated from the spontaneous PD and short-circuit current (*I*_sc_). After a 30-min equilibration period on Ussing chambers, TER was recorded at 1-min intervals over a 60-minute period and then averaged to derive the basal TER values for a given animal.

### Mucosal-to-serosal fluxes of FITC-labeled dextran

Mucosal barrier properties were assessed at the same time as TER measurements. After a 15-minute period on Ussing chambers, 0.25 mM FITC Dextran (FD4; 4 kDa, Sigma-Aldrich, St. Louis, MO) was placed on the mucosal side of Ussing chamber-mounted tissues. After a 15-min equilibration period, 50 μL samples (in triplicate) were taken from the serosal side of tissues at 30-min intervals and transferred into a 96-well assay plate. The FD4 fluorescence intensity of each sample was measured by fMax Fluorescence Microplate Reader (Molecular Devices, Sunnyvale, CA) and concentrations were determined from standard curves generated by serial dilution of FD4. FD4 flux was measured for 60 min and presented as the rate of FD4 flux (μg/min∙cm^2^)

### Histological analyses

Ileal and colonic tissues were taken immediately after euthanasia and stored in 10% neutral buffered formalin until processing for routine histological evaluation. Paraffin-embedded intestinal samples were sectioned (5 μm) and stained with hematoxylin and eosin for histological analysis under a light microscope. For quantification of mast cells, intestinal tissues (n = 4 animals/treatment group) were processed for immunohistochemical staining for porcine mast cell tryptase. In brief, 10% NBF-fixed paraffin-embedded ileum and colon tissue sections were processed for routine immunohistochemistry and epitope retrieval and then stained with Rabbit Polyclonal anti-Mast Cell Tryptase (Santa Cruz Biotechnology, Santa Cruz, CA; 1:200 dilution) in normal antibody diluent (NAD-Scytek) for 30 minutes. Tissue sections with then treated with Rabbit anti–Farma Promark^™^ Micro-Polymer (Biocare) for 30 minutes followed by reaction development with Romulin AEC^™^ (Biocare) 10 minutes and Hematoxylin counterstain for 5 minutes. Mast cells were counted at 40x magnification using a micrometer grid fitted within an eyepiece. For each tissue slide, 80 non-overlapping areas (total surface area 4.8 mm^2^) were counted to estimate mucosal and submucosal mast cell numbers, expressed as cells/mm^2^. Photomicrographs were acquired with 20× and 40× magnification at a resolution of 1360 × 1024 using imaging software (Olympus DP2-BSW version 2.2), a high-resolution digital camera (Olympus DP72) affixed to a clinical light microscope (Olympus BX45). Prior to imaging, the system was calibrated at each magnification using a stage micrometer. Measurements were taken using the arbitrary line tool and exported into a spreadsheet program (Excel2010, Microsoft). Villi were measured for villus height and crypt depth by using the 20× objective. For each histological slide prepared for each pig, we located four different areas on the slide within the 20x field view that contained at least three, well-oriented villi based on the criteria that 1) the entire crypt and villi were captured in cross section and 2) the central lacteal was present. Therefore a minimum of 12 individual villi measurements per pig were taken and then averaged to derive the mean villi height for each pig. All histopathology data were analyzed as n = 4 pigs per treatment.

### Gene expression analysis

Immediately after euthanasia, the segments of ileum and colon were removed from the pig, cut open longitudinally and rinsed generously with 0.87% saline to remove any digesta prior to sample collection. The scrapings of the exposed mucosa were collected, immediately flash frozen in liquid N_2_ and stored at -80°C until gene and protein analysis. Total RNA samples were isolated from frozen intestinal mucosal scrapings using the Qiagen RNeasy Mini kit. First-strand cDNA was synthesized from 3 μg RNA using Thermo Scientific Maxima First Strand cDNA Synthesis Kit for RT-qPCR with dsDNase (Thermo Scientific, Waltham, MA) according to the manufacturer’s instructions. Semi-quantitative, real-time PCR was used to determine the relative quantities of transcripts of the genes of interest. The genes encoding the 60S ribosomal subunit (*RPL4*) and beta-actin (*ACTB*) were selected and validated as suitable internal reference genes [37]. The relative gene expressions of corticotropin releasing factor (CRF) family genes CRF, *Ucn2*, CRF receptor 1 (*CRF1*), CRF receptor 2 (*CRF2)*, and CRH binding protein (*CRHBP*); nutrient transporters sodium-glucose linked transporter 1 (*SGLT1*), glucose transporter 2 (*GLUT2*), fructose transporter (*GLUT5*), excitatory amino-acid transporter 3 (*EAAT3*), neutral amino acid transporter (*B0AT1*), and neutral and cationic amino acid transporter (*ATB0+*); mast cell tryptase (*MCT7*) and mast cell chymase (*CMA1*), pro-inflammatory cytokines, tumor necrosis factor alpha (*TNFA*), interleukin 1 beta (*IL1β*), interleukin 6 (*IL6*), interleukin 8 (*IL8*), interleukin 10 (*IL10*) and interferon gamma (*IFNG*) were determined. Primer sequences for all genes are provided as a Supporting Information file (S1 Table). All PCR reactions were subjected to a melt curve analysis to validate the absence of nonspecific products. The data are presented as 2^-ΔΔCT^ in gene expression relative to control group, normalized to the geometric mean of *RPL4* and *ACTB* [37] before statistical analysis.

### ELISA assays

*Serum cortisol*: One pig per pen (n = 6 pigs /treatment) was selected at random to obtain a blood sample via jugular venipuncture at the same time (0800 to 0900 am) on d 0 and 7 of the CSS experiment. Serum was obtained by centrifugation and stored at -80°C until analysis. The serum cortisol level was measured by the commercial ELISA kit (Enzo Life Sciences, Inc., Farmingdale, NY).

*Ileal mucosal cytokines*: Protein was extracted from ileal mucosal homogenates and protein quantified with a BSA assay. The intestinal protein samples were examined in triplicate for cytokine concentrations (TNFα, IL1β and IL8) using a commercial ELISA kit (R&D Systems, Minneapolis, MN).

### Statistical analysis

Data were analyzed by using the Student’s t-test in SAS (version 9.4; SAS Institute). For statistical analysis of performance data, the pen served as the experimental unit (n = 6 pens/experimental treatment). For other data (physiology, immune measurements, cortisol, etc.) the individual animal served as the experimental unit (n = 4–6 per treatment). Differences were considered significant at P ≤ 0.05, and tendencies were discussed at P > 0.05 to 0.1. Values reported are means ± SE.

## Results

### Chronic social stress impairs growth rate and feed efficiency and alters nutrient transporter function and expression

**Fig 1:**
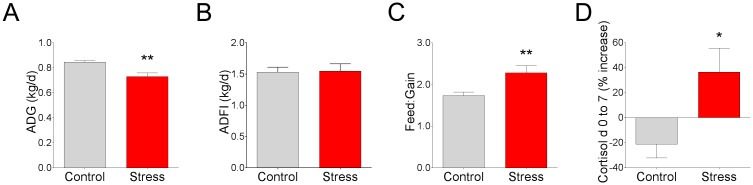
Effects of chronic social stress on growth performance and serum cortisol in pigs. Pigs (22 ± 0.54kg, Yorkshire cross breed) were subjected to 7 d of chronic social stress by reducing floor space (0.17 m^2^/pig) and mixing with new pigs. Control pigs were housed at a floor space allowance of 0.33 m^2^/pig without mixing pigs. **(A)** Average daily gain (ADG). **(B)** Average daily feed intake (ADFI). **(C)** The feed:gain ratio. **(D)** Serum cortisol calculated as a % change between d0 and d7 of the study. Values are means ± SE (n = 6 per treatment). ** indicates significant difference, P ≤ 0.01; * indicates significant difference, P ≤ 0.05 by Student’s t-test.

In the present study, we observed a 0.116 kg/d lower in average daily gain (ADG) in CSS pigs compared with controls (Fig 1); however, significant differences in feed intake (ADFI) were not observed (Fig 1). As a result, the feed:gain ratio was increased by 22% in stressed pigs (Fig 1). Relative to d 0 of the trial, serum cortisol levels decreased 2.0 ± 1.1 ng/mL for control animals and increased by 2.70 ± 2.1 ng/mL in CSS animals when measured following 7 d CSS (Fig 1), indicating increased HPA axis activity in CSS pigs thus and confirming the increased stress levels.

**Fig 2:**
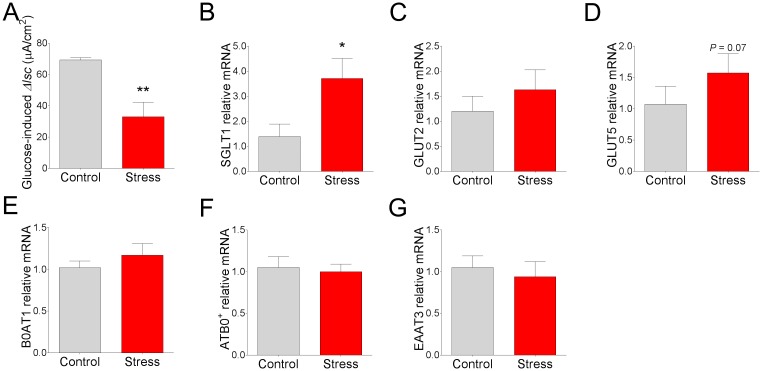
Effects of chronic social stress in pigs on intestinal glucose transporter activity and transporter expression. **(A)** Glucose-induced Δ*I*_sc_. Relative mRNA expression of **(B)** SGLT-1, **(C)** GLUT2, **(D)** GLUT5, **(E)** B0AT1, **(F)** ATB0^+^, and **(G)** EAAT3. All values are relative to the expression levels measured in control pigs. Values are means ± SE (n = 6 per treatment). ** indicates significant difference, P ≤ 0.01; * indicates significant difference, P ≤ 0.05; P = 0.07 by Student’s t-test.

**Fig 3:**
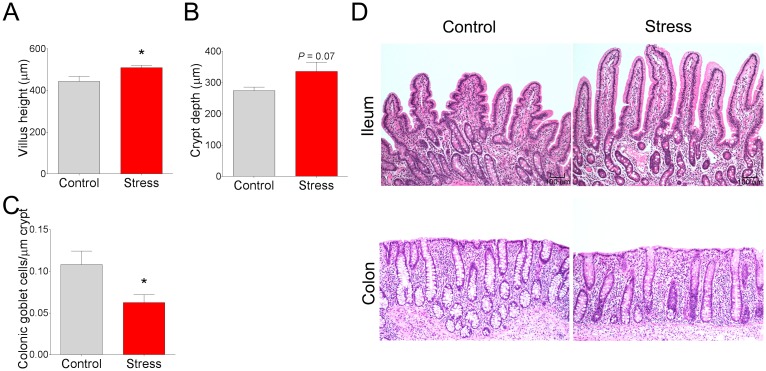
Histological appearance and intestinal morphology in pigs subjected to chronic social stress. **(A)** Ileal villus height. **(B)** Ileal crypt depth. **(C)** Goblet cell count in the colon. **(D)** Representative samples from histological stained sections showed no differences in histologic inflammation but stressed pigs had higher ileal villus height and reduced colonic goblet cells. Values are means ± SE (n = 4 per treatment). * indicates significant difference, P ≤ 0.05; P = 0.07 (B) by Student’s t-test.

To determine whether nutrient transporter activity and expression was altered by CSS in pigs, we mounted ileal mucosa from control and CSS pigs on Ussing chambers and measured transepithelial short circuit current (*I*_sc_) responses to luminal glucose addition, an index of the apical Na^+^-glucose linked transporter 1 (SGLT-1) activity. Luminal glucose induced a significant elevation in *I*_sc_ in control and CSS ileum; however, the response in CSS pigs was 52% lower compared with control pigs (Fig 2), suggesting an impaired glucose transport mechanism in CSS pigs. We next investigated whether the functional changes in SGLT-mediated glucose transport function were associated with stress-induced changes in transporter expression or villus morphology. qPCR revealed that expression for SGLT-1 was upregulated (by 1.7 fold; Fig 2) in CSS pig ileum, compared with controls. No significant differences in gene expression of GLUT2, the major transporter involved in basolateral glucose uptake, was observed (Fig 2). However, a trend (P = 0.07) for an increased gene expression of the fructose transporter, GLUT5, was observed (Fig 2) in CSS pigs, compared with controls. As a comparison to sugar transporters, we also assessed the gene expression for amino acid transporters ATB0^+^, EAAT3, and B0AT1 and found no significant differences between control and CSS pigs (Fig 2). Histo-morphometric analysis of ileum from pigs revealed that CSS pigs exhibited increased ileal villus height (Fig 3). The means for crypt depth were numerically increased (Fig 3; P = 0.07), but not significantly different compared with controls. Overall, these data suggest that while SGLT-1 gene expression is upregulated along with increase villus lengths in CSS pigs, a significantly compromised SGLT-1 transporter function was observed.

### Chronic social stress in pigs impairs intestinal barrier function

**Fig 4:**
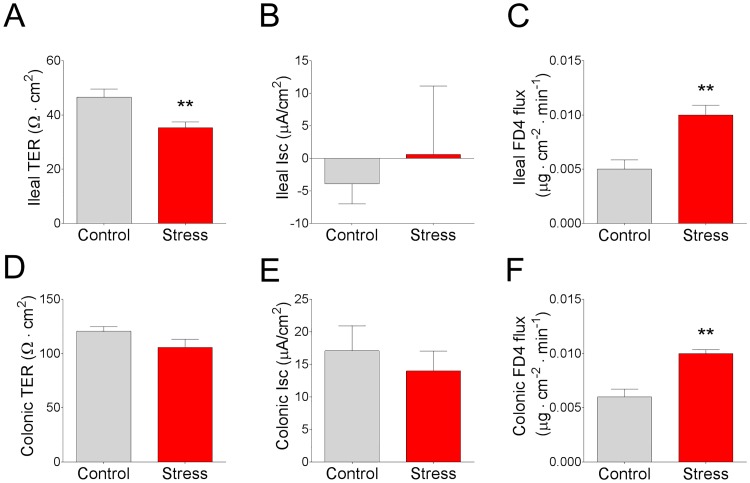
Effects of chronic social stress in pigs on ileal and colonic TER, *I*_sc_, and FD4 flux rates. **(A)** Ileal TER. **(B)** Basal ileal *I*_sc_. **(C)** Ileal FD4 flux rate. **(D)** Colonic TER. **(E)** Basal colonic *I*_sc_. **(F)** Colonic FD4 flux rate. Values are means ± SE (n = 6 per treatment). ** indicates significant difference, P ≤ 0.01.

To determine whether CSS in pigs influenced intestinal permeability, we assessed transepithelial TER and mucosal-to-serosal flux of FD4 in ileal and colonic mucosa mounted on Ussing chambers. Compared with non-stressed control pigs, CSS pigs exhibited reduced TER in the ileum (Fig 4) while no significant differences in TER were observed in the colon (Fig 4). In CSS pigs, paracellular permeability, assessed as FD4 flux rate, was elevated in the ileum (by 0.8 fold; Fig 4) and colon (by 0.7 fold; Fig 4) compared with control pigs. No significant differences were observed in baseline *I*_sc_ values between experimental groups (Fig 4).

### Intestinal CRF system gene expression in pigs subjected to chronic social stress

**Fig 5:**
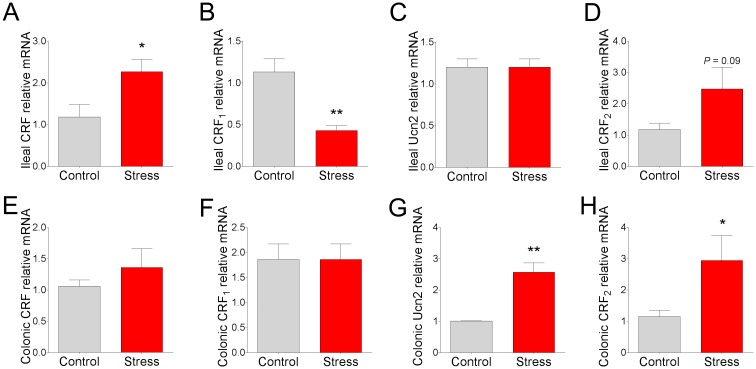
CRF, Ucn2, and CRF receptor expression in ileal and colonic mucosa in pigs subjected to chronic social stress. Relative mRNA expression of **(A)** ileal CRF, **(B)** ileal CRF_1_, **(C)** ileal Ucn2, **(D)** ileal CRF_2_, **(E)** colonic CRF, **(F)** colonic CRF_1_, **(G)** colonic Ucn2, and **(H)** colonic CRF_2_ in pigs subject to chronic social stress. All values are relative to the expression levels measured in control pigs. Values are means ± SE (n = 6 per treatment). ** indicates significant difference, P ≤ 0.01; * indicates significant difference, P ≤ 0.05; P = 0.09 (D) by Student’s T-test.

We next determined whether CSS in pigs altered stress signaling pathways in the mucosa by measuring gene expression of CRF-family ligands CRF and Ucn2 and their respective receptors, CRF_1_ and CRF_2_. In the ileum, CRF mRNA was upregulated in CSS pigs (by 1 fold; Fig 5) compared with control pigs. However no significant differences in CRF gene expression between treatment groups were observed in colonic mucosa (Fig 5). Gene expression for Ucn2, the CRF_2_–specific ligand, was not altered by CSS in the ileum (Fig 5), but was upregulated (by 1.5 fold) in colonic mucosa in CSS pigs (Fig 5). In the ileum, CRF_1_ mRNA was down-regulated (by 62%; Fig 5) in CSS pigs whereas no changes were observed in the colon (Fig 5). There was a trend (P = 0.09) for upregulated CRF_2_ mRNA in ileal mucosa from CSS pigs (Fig 5) and a significant CRF_2_ upregulation (by 1.5 fold; Fig 5) observed in the CSS colonic mucosa. Together, these data indicate a differential and site-specific regulation of gene expression of the CRF system in response to CSS in pigs.

### Intestinal mucosal cytokine expression in response to chronic social stress in pigs

Upon histological examination, there were no remarkable alterations in the appearance of the ileal and colonic mucosa with regards to lamina propria cellularity, edema, hyperemia, or epithelial changes between the treatment groups (Fig 3). There was, however, a reduction in goblet cell numbers observed in the colon of CSS pigs (Fig 3).

**Fig 6:**
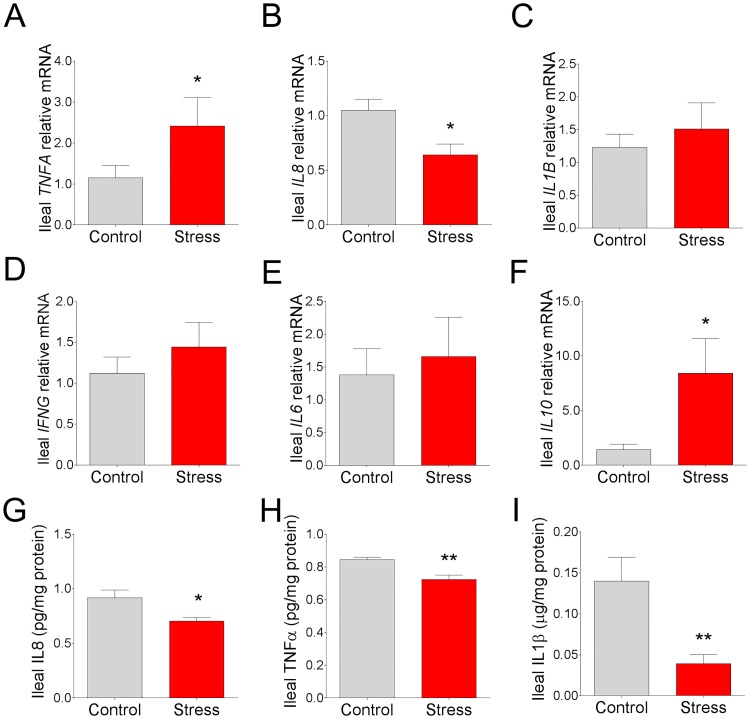
Effects of chronic social stress on ileal mucosal cytokine gene and protein expression. Relative mRNA expression of **(A)** *TNFA*, **(B)** *IL8*, **(C)** *IL1B*, **(D)** *IFNG*, **(E)** *IL6*, and **(F)** *IL10* in ileal mucosa from pigs subjected to 7 d chronic social stress. All values are relative to the expression levels measured in control pigs. Ileal mucosal concentrations of **(G)** IL-8, **(H)** TNFα and **(I)** IL1β from pigs subjected to 7 d chronic social stress. Values are means ± SE (n = 6 per treatment). ** indicates significant difference, P ≤ 0.01; * indicates significant difference P ≤ 0.05. Student’s T-test.

In the ileum, there was a 1-fold upregulation in *TNFA* mRNA (Fig 6; P < 0.05) while *IL8* mRNA was downregulated (by 39%) (Fig 6; P < 0.05) in stressed pigs compared to control pigs. No differences in mRNA expression were observed with *IL1B*, *IL6 or IFNG* between experimental groups (Fig 6). There was a marked upregulation (by 4.9 fold) of *IL10* mRNA observed in the CSS ileum (Fig 6) compared to the ileum of control pigs. ELISA assays for select cytokines were conducted for comparison with qPCR results. IL-8 mucosal protein levels (Fig 6) were lower (by 23%) in CSS ileum, which was in agreement with ileal *IL8* mRNA expression. In contrast to mRNA expression levels, TNFα protein levels were lower (by 14%) in the CSS ileum (Fig 6). IL1-β protein levels were markedly lower (by 72%) in CSS ileum compared with non-stressed controls (Fig 6).

**Fig 7:**
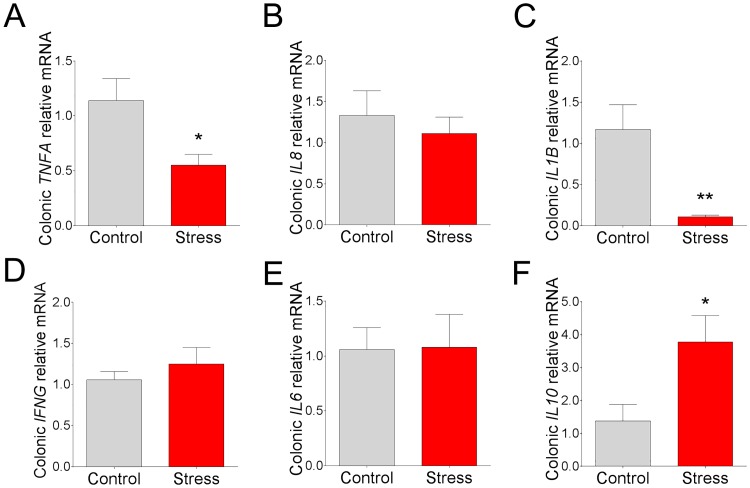
Effects of chronic social stress on colonic mucosal cytokine gene expression. Relative mRNA expression of **(A)** *TNFA*, **(B)** *IL8*, **(C)** *L1B*, **(D)** *IFNG*, **(E)** *IL6*, and **(F)** *IL10* in colonic mucosa from pigs subjected to 7 d chronic social stress. All values are relative to the expression levels measured in control pigs. Values are means ± SE (n = 6 per treatment). ** indicates significant difference, P ≤ 0.01; * indicates significant difference, P ≤ 0.05 by Student’s t-test.

In the colon, a downregulation of *TNFA* (by 52%) and *IL1B* (by 91%) mRNA was observed in CSS pigs (Fig 7). No significant differences were observed with regards to *IFNG*, *IL6* or *IL8* gene expression (Fig 7). Similar to the ileum, there was an upregulation of *IL10* mRNA (by 1.7 fold; Fig 7) observed in the CSS colon.

**Fig 8:**
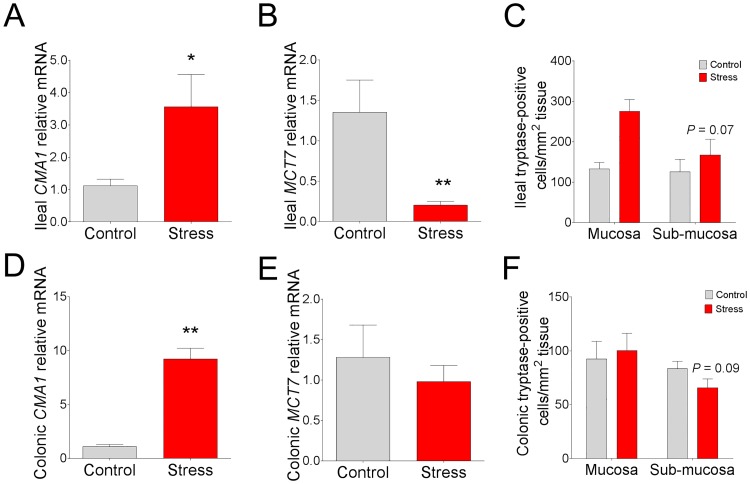
Effects of chronic social stress in pigs on mast cell chymase and tryptase mRNA expression and mast cell tryptase staining in ileum and colon. Relative mRNA expression of **(A)** ileal *CMA1* and **(B)** ileal *MCT7*. **(C)** Ileal submucosal mast cell tryptase-positive counts. Relative mRNA expression of **(D)** colonic *CMA1* and **(E)** colonic *MCT7*. **(F)** Colonic submucosal mast cell tryptase-positive counts. mRNA values are relative to the expression level measured in control pigs. Values are means ± SE (n = 6 per treatment). ** indicates significant difference, P ≤ 0.01; * indicates significant difference, P ≤ 0.05; P = 0.07 (C), P = 0.09 (F) by Students T-test.

Because mast cells are a critical innate immune cell known for their involvement in orchestrating mucosal immune responses and mediating stress-induced alterations in GI epithelial barrier function we measured the mRNA gene expression for mast cell protease genes in the ileum and colon in CSS pigs. In pigs exposed to 7 d CSS, mucosal mast cell mRNA for mast cell chymase (*CMA1*) was markedly upregulated in the ileum (by 2.2 fold; Fig 8) and colon (by 7.5 fold; Fig 8), compared with control pigs. In contrast, mRNA expression for mast cell tryptase (*MCT7*) was significantly downregulated in the ileum (by 85%; Fig 8). The mean value for *MCT7* mRNA was lower (by 23%) in the colon from CSS pigs; however, this was not statistically significant from control pigs (Fig 8). To gain more insight into the down-regulation of *MCT7* observed in CSS pigs, we performed immunohistochemical staining for mast cell tryptase in the mucosa and submucosa of control and CSS pigs. The number of tryptase-positive cells in the ileal and colonic mucosal region were not different between control and CSS pigs; however, although not significant, there was a numerical reduction in the number tryptase-positive cells in both the ileum (P = 0.07) and colonic (P = 0.09) submucosa of CSS pigs (Fig 8). Taken together, these data indicate that CSS has differential effects on cytokine and mast cell gene and protein expression in the ileum and colon in pigs, characterized predominantly by a marked down-regulation of pro-inflammatory cytokines and *MCT7* and an upregulation of *IL10* and *CMA1*.

## Discussion

Stress is a major factor in the onset of GI disorders in humans and animals. The precise mechanisms by which psychosocial stress increases GI disease susceptibility remain incompletely understood. Results from the present study demonstrated that chronic stress in pigs resulted in significant alterations in multiple aspects of GI function including impairment of epithelial barrier and nutrient transport function, diverse effects on CRF system ligand and receptor expression, and immune suppression. Together these data provide insight into the impact of chronic stress on GI function in a large animal model.

In the present study, pigs subjected to CSS exhibited a reduced growth rate and feed efficiency that was not associated with reduced feed intake. The results are in line with Hyun et al. (2000) who conducted similar experiments (mixing and crowding stress) in pigs [6]. The mechanism for stress-induced impairment in feed efficiency is poorly understood but our findings, along with that of Hyun et al. (2000) provide evidence that mechanisms other than reduced feed intake contribute significantly to growth performance reductions in stressed animals. Ileal glucose-induced Δ*I*_sc_ measurements conducted in Ussing chambers revealed a significantly compromised SGLT1 function. Despite the reduction in SGLT1-mediated glucose transport in CSS ileum, SGLT1 gene expression was upregulated along with increased ileal villus height. The mechanisms regulating the divergent effects of CSS on glucose nutrient transporter function, transporter expression, and villus-crypt morphology are not fully understood. Increased SGLT1 expression and increased villus length proliferation observed in the present study may represent a compensatory mechanism to attempt to preserve nutrient transporter function during times of chronic stress when transporter function is compromised. Similar to our findings, Boudry et al. [38] reported that, in a model of chronic stress (5 d of water avoidance stress) in rats, jejunal SGLT1 function as measured by glucose uptake across isolated brush border membrane vesicles was reduced in stressed rats. However, in contrast to findings in the present study, the authors reported no changes in SGLT1 expression and reported an increased activity and protein expression of GLUT2 [38]. Similar to the present study, Lee (2013) reported that chronic restraint stress in rats (4 hr daily restraint stress for 8 weeks) increased the expression SGLT-1 in the jejunum and ileum compared with controls [39]. The mechanisms responsible for impaired SGLT1 function in CSS pigs are unknown. Stress can influence a number of GI systems involved in the regulation of nutrient transport mechanisms including ENS, immune and epithelial changes. Immune/inflammatory mediators and (or) infectious challenges have been shown to alter the expression and function of SGLT1 transport in the intestine. For example, elevated cytokines such as IL-6, IL-1α and IL-8, but not IL-10, were shown to improve the absorption of glucose *ex vivo* in rabbit jejunal tissue [40]. In pigs, experimental infection with porcine reproductive and respiratory syndrome virus (PRRSv) increased jejunal glucose transporter activity while infection with porcine endemic diarrhea virus (PEDv) reduced jejunal glucose transport activity when measured 21 d post-challenge [41] with no effect on SGLT1 expression. In the present study, we observed down-regulation of pro-inflammatory cytokines and an increase in intestinal mast cell activity, but the role of these factors in modulating SGLT1 transport under chronic stress remains to be elucidated. It is also possible that stress-induced impairment in barrier function in CSS pigs could have also contributed to impaired nutrient transporter function. Impaired barrier function and loss of tight junctions influences polarization and basolateral/apical separation of ion and nutrient transporters (fence function) which is important for the establishment of effective electrochemical gradients to drive epithelial transport mechanisms such as SGLT1-mediated glucose transport. In summary, findings from the present study demonstrated an impaired SGLT1 function in pigs subjected to CSS, which may represent an important mechanism driving impaired reduced weight gain and feed efficiency observed in pigs under chronic stress conditions.

The CRF system is the major stress system regulating central and peripheral responses to stress. While CRF system responsiveness is critical for survival and stress-coping mechanisms, excessive activation or dysregulation of this system is central to disease pathogenesis, including stress-induced GI disorders such as IBS. In the present study, pigs subjected to CSS exhibited significant and diverse changes in CRF system-related genes in the ileum and colon. While CRF gene expression was increased in the ileum of CSS pigs, no significant changes were observed in the colon. In contrast, the CRF_2_ receptor ligand Ucn2 was significantly upregulated only in the colon, suggesting a differential synthesis and regulation of CRF ligands in the ileum and colon in chronically stressed pigs. There were also differential effects of CSS with regards to CRF_1_ and CRF_2_ receptor gene expression, where CRF_1_ expression was decreased in the ileum only of CSS pigs, while CRF_2_ gene expression was upregulated in the colon but not ileum (P = 0.09) following CSS. Down-regulation of ileal CRF_1_ receptor expression could reflect receptor-dependent endocytosis in the presence of high ileal CRF levels, which has been shown to be a mechanism of regulation of CRF_1_ [42]. In contrast to our findings, 15 d of crowding stress in rats was also shown to increase CRF_1_ protein expression [43]. The functional relevance of the differential and site-specific expression patterns of the CRF and Ucn2 and their receptors observed in the present study remain to be elucidated. In general, it has been shown that several stress-induced pathophysiologic changes in the intestine, including secretion, motility, and visceral hypersensitivity, are mediated via CRF_1_ receptors [31, 33] while CRF_2_ signaling has been shown to be inhibitory to CRF-mediated motility and visceral hypersensitivity [27, 32]. While CRF_2_ has been shown to play an inhibitory role in motility disturbances, evidence exists for a role of CRF_2_ in mediating GI inflammatory responses as demonstrated in a *Clostridium difficile* toxin A-mediated inflammation and in colonic epithelial cells [44]. In summary, data here demonstrate a significant influence of CSS on intestinal CRF system expression in a site-specific manner in the porcine intestine. These findings may reflect a differential role of CRF system ligands and their receptors in modulating responses in different sites of the GI tract in response to CSS.

Stress has long been known to influence the systemic immune response. However, less is known about how stress influences GI mucosal immune responses. In rodent models, chronic stress has been shown to induce predominantly inflammatory changes in the GI tract, including inflammatory cell infiltration and upregulation of cytokines [19, 39]. However, findings from the present study in pigs subjected to CSS revealed a predominantly immunosuppressive influence of CSS, characterized by a significant down-regulation of pro-inflammatory cytokines including TNFα, IL1β, and IL-8. This finding was particularly remarkable given the increased intestinal permeability in CSS pigs, which would be generally associated with inflammation due to increased luminal antigen exposure [45]. The mechanisms driving down-regulated cytokine expression in CSS pigs are unknown; however, a potentially important finding in the present study was the robust upregulation of IL10 gene expression in both CSS ileum and colon. Comparable with our findings, elevated IL10 was observed in mice subjected to 5-day chronic restraint-stress protocol [46]. IL10 is well known for its role in the suppression of inflammatory cytokines including IL1, IL6, IL12, and TNF, and immunosuppression as reviewed previously [47] and therefore could be a potential mechanism for CSS-induced down-regulation of pro-inflammatory cytokines in the present study. The disease implications for the suppressed pro-inflammatory cytokines induced by CSS remains to be elucidated. Compromised host immune responses could impair mucosal immune regulation and defense against opportunistic or invading pathogens. In line with this concept, we previously reported that early life stress in pigs resulted in chronic intestinal barrier dysfunction, impaired mucosal immune response, and increased severity of clinical disease induced by an infectious challenges with enterotoxigenic *Escherichia coli* [9].

Increased mast cells play an important role in stress-induced GI disorders such as IBS [48, 49]. Stress-induced mast cell activation induces the release of pre-formed granule mediators including proteases (chymase and tryptase) and histamine, which cause profound changes in intestinal function such as intestinal permeability, hypersecretion, inflammation and visceral hypersensitivity [8, 12]. In the present study, ileum and colon from CSS pigs exhibited an up-regulation of mast cell chymase gene (*CMA1*) expression, while in contrast mast cell tryptase gene (*MCPT7*) expression and tryptase-positive cell numbers were decreased. These findings indicate that GI mast cell chymase and tryptase gene expression is divergently altered in CSS pigs. The reason for the divergent expression patterns of mast cell chymase and tryptase is unclear but suggests a shift in mast cell protease synthesis from tryptase to chymase in response to CSS. Mast cells are a highly plastic cells that are capable of changing their protease composition in response to changing environmental cues including bacterial products, inflammatory stimuli, and cytokines [50, 51]. Mast cells are well known for their role in inflammation in diseases such as allergy and pathogen-mediated immune responses [52]. In contrast, mast cell activation can also be immunosuppressive. Specifically, mast cell chymase can degrade inflammatory cytokines, which has been shown to limit inflammation and improve survival in a model of sepsis [53]. In addition, histamine was shown to exert potent immunosuppressive effects by interacting with and inhibiting dendritic cells [54]. In contrast, mast cell tryptase has been shown to be predominantly inflammatory in the gut [55, 56]. In a study by Chan et al., it was shown that mast cell-derived IL10 was upregulated in a murine model of urinary tract infections, which was shown to suppress immune activation and tolerance in the bladder and was hypothesized to contribute to impaired adaptive immune responses and bladder bacterial persistence [57]. Given the diverse capabilities of mast cell products mentioned above, downregulation of tryptase and upregulation of chymase, along with upregulation of IL10 could be an important mast cell-mediated adaptive response to limit inflammatory responses during chronic stress, especially under conditions of impaired barrier function.

In summary, data presented here in a model of CSS in pigs confirmed common mechanisms of stress-induced pathophysiology observed in similar chronic stress paradigms in rodents including impaired barrier and nutrient transport, activation of the enteric CRF system, and upregulation of mast cell activity. The present study also revealed new and contrasting findings with the rodent literature with regards to the chronic stress-induced modulation of intestinal immune responses which was predominantly characterized by immune suppression and upregulation of IL10. These data highlight that, while many stress-induced alterations in GI function are consistent across rodents, pigs, and humans, and across different models and environmental factors, some responses such as immune responses can vary significantly.

## Supporting information

S1 Table Primers and TaqMan probes of gene of interest in pigs. (PDF)
