# Supplementary material for: Chronic social stress in pigs impairs intestinal barrier and nutrient transporter function, and alters neuro-immune mediator and receptor expression
Source: PLoS One. 2017 Feb 7;12(2):e0171617. doi: 10.1371/journal.pone.0171617 (PMC5295718; doi:10.1371/journal.pone.0171617)
Supplement: S1 Table — (PDF) [file pone.0171617.s001.pdf]

S1 Table.

Gene symbol		Sequence
ACTB	Forward	GAAGCTCAGTCGGGCTTCTC
	Reverse	ATGTCGACGTCGCACTTCAT
RPL4	Forward	AGCGAATGAGAGCTGGTAAAG
	Reverse	TTACGCCAAGTGCCATAGAG
MCT7	Forward	CTGAGATGCCTCGACCAATAC
	Reverse	TCCGTTGACCTCGTCATAGTA
CAM1	Forward	TTCACCCGGATCTCCCATGA
	Reverse	GAGACACACACTCGGTCTGG
IFNG	Forward	CCAGGCCATTCAAAGGAGCAT
	Reverse	TCAGTTTCCCAGAGCTACCA
TNFA	Forward	TGGGCTGTACCTCATCTACT
	Reverse	CACTGAGTCGATCATCCTTCTC
IL1B1	Forward	GAAAGCCCAATTCAGGGACC
	Reverse	GGCGGGTTCAGGTACTATGG
IL6	Forward	TAAGGGAAATGTCGAGGCCG
	Reverse	TTGTGTTCTTCATCCACTCGT
IL8	Forward	GACCCCAAGGAAAAGTGGGT
	Reverse	GAGGCAAGAAGACTTGTGAATGC
IL10	Forward	TCGGCCCAGTGAAGAGTTTC
	Reverse	GGAGTTCACGTGCTCCTTGA
CRH	Forward	CCCATCTCGCTGGATCTCAC
	Reverse	CCAAACGCACCGTTTCACTT
Ucn2	Forward	GTCACTGCCATGTGGTGTCA

	Reverse	GACATGGCAACATCCAGGCT
CRHR2	Forward	CCGCAATGCCTACCGAGAAT
	Reverse	TCATCCAAAATGGGCTCGCA
CRHBP	Forward	GCTGGGAGGAACTGGATTGG
	Reverse	CTGGCGGTACTCAAAAGTCAC
SGLT1	Forward	GTGCAGTCAGCACAAAGTGG
	Reverse	GTTCACTACTGTCCGCCACA
GLUT2	Forward	TTTTGGGTGTTCCGCTGGAT
	Reverse	GAGGCTAGCAGATGCCGTAG
ACTB	TaqMan probe	Ss03376563_uH
GLUT5	TaqMan probe	Ss03377332_u1
B0AT1	TaqMan probe	Ss03376931_u1
ATB0+	TaqMan probe	Ss03376400_u1
EAAT3	TaqMan probe	Ss03373659_m1
CRHR1	TaqMan probe	Ss03373288_g1
